# Swappable hydrogen storage for road vehicles

**DOI:** 10.1016/j.isci.2026.117655

**Published:** 2026-09-26

**Authors:** William Forshaw, Timothy Mays, Alfred Hill, Tom Fletcher

**Affiliations:** 1 Department of Chemical Engineering, University of Bath, Bath, UK; 2 Department of Mechanical Engineering, University of Bath, Bath, UK

**Keywords:** hydrogen, refuelling, swappable, distribution, automotive, infrastructure, fuel, modular

## Abstract

Despite significant advancements in hydrogen storage technology, a challenge remains to provide affordable hydrogen refueling infrastructure for road vehicles. This article suggests a swappable hydrogen tank method for distributing and dispensing fuel. Swappable refueling may reduce the upfront cost of hydrogen refueling stations, reduce the energy consumed to dispense high-pressure hydrogen and increase the efficiency of distribution over standard tube trailers. In the analysis, current systems for transporting and dispensing gaseous hydrogen fuel account for a significant amount of energy consumed calculated at 23.8 MJ kg^−1^. In contrast, the swappable delivery system consumes only 9.7 MJ kg^−1^; because hydrogen is compressed at the point of production, transport is 5x more efficient and removes the need for gas handling equipment at every station. This 60% energy saving and the anticipated equipment reductions present a case for an alternative refueling method to address the issues facing hydrogen vehicles.

## Highlights

• Swappable refueling may improve energy efficiency up to 60% vs. current stations
• Swappable refueling could decrease the equipment required to operate a station
• Transporting hydrogen at higher pressures could reduce fuel deliveries by 5-fold

## Graphical abstract

**Figure:**
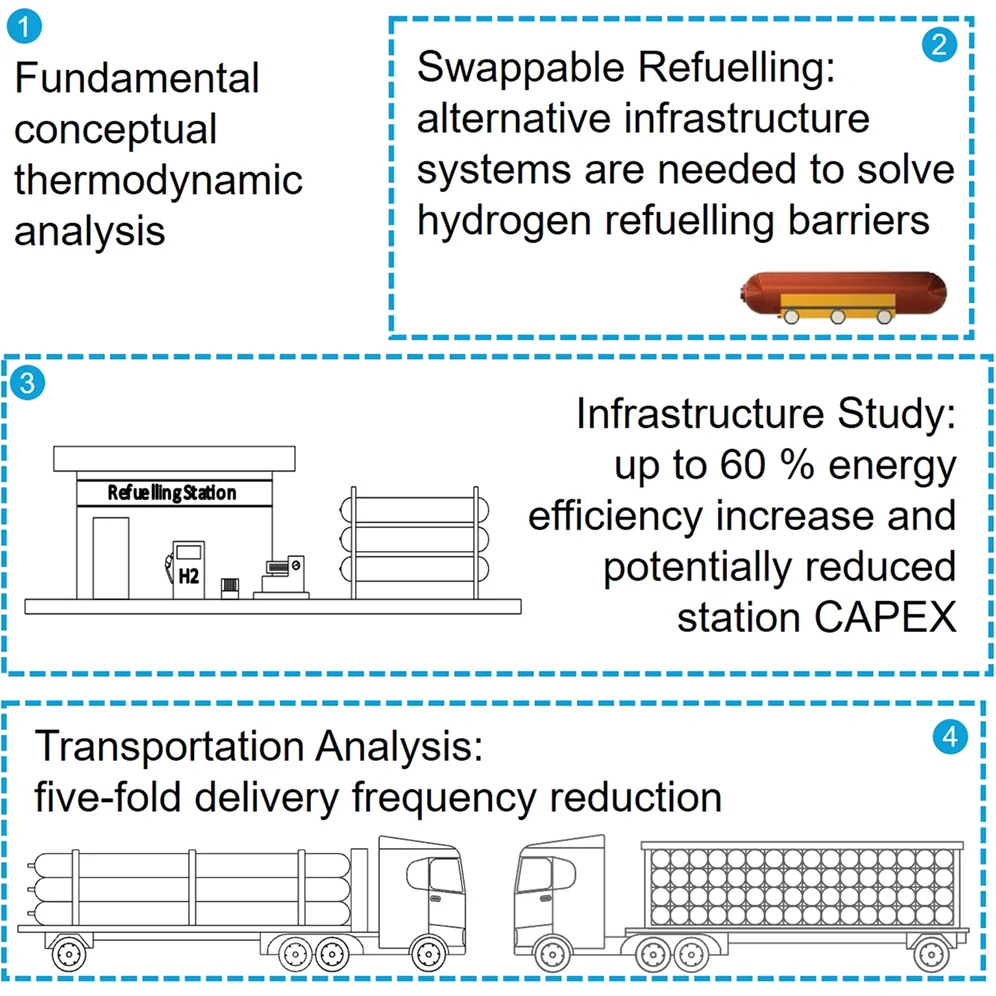

## Teaser

Applied sciences; Energy engineering; Energy storage

## Introduction

To facilitate a sustainable shift from fossil fuels for road vehicles, there needs to be refueling infrastructure available. However, this leads to a “chicken and egg” scenario of whether users or infrastructure should come first. Customers are otherwise hesitant to adopt an alternative fuel vehicle, sources such as Pevec et al.^1^ found that the “availability of refueling infrastructure” was currently a major concern for vehicle end-users. Meanwhile current station design presents a risky investment, as insufficient demand exists to make stations profitable.^2^ Currently, the refueling speed of hydrogen refueling stations (HRSs) is comparable to that of fossil fuels however the high cost of fuel, equipment and lack of infrastructure has slowed the rollout of hydrogen vehicles.^3^ This presents a window of opportunity to influence the design of hydrogen refueling infrastructure and adopt industry wide standardization before vehicles roll off the production line and while infrastructure projects are still on the drawing board.

Low carbon hydrogen as a fuel it offers a high gravimetric energy density of 120 MJ kg^−1^ (lower heating value) but over a hundred times less volumetric density than gasoline or diesel.^4^ To overcome this, hydrogen is compressed to provide a practical volumetric density but this process brings several challenges. Firstly, higher pressures require a stronger container which can increase the weight and cost of the fuel system. Secondly, an increase in pressure does not provide a linear increase in density which further limits the amount of fuel that can be practically stored safely. Past ∼100 bar the behavior of hydrogen deviates significantly from an ideal gas. Above 500 bar, the diminishing returns from increased compression become apparent with nearly a 50% deviation from the ideal. Figure 1 illustrates this, the compression energy required per kg of normal hydrogen increases dramatically meaning additional work is required, reducing energy efficiency. Therefore, a balance must be struck, where higher pressures deliver greater storage densities against the additional equipment cost and energy consumption which is an important factor when looking from an infrastructure scale perspective.

**Figure 1:**
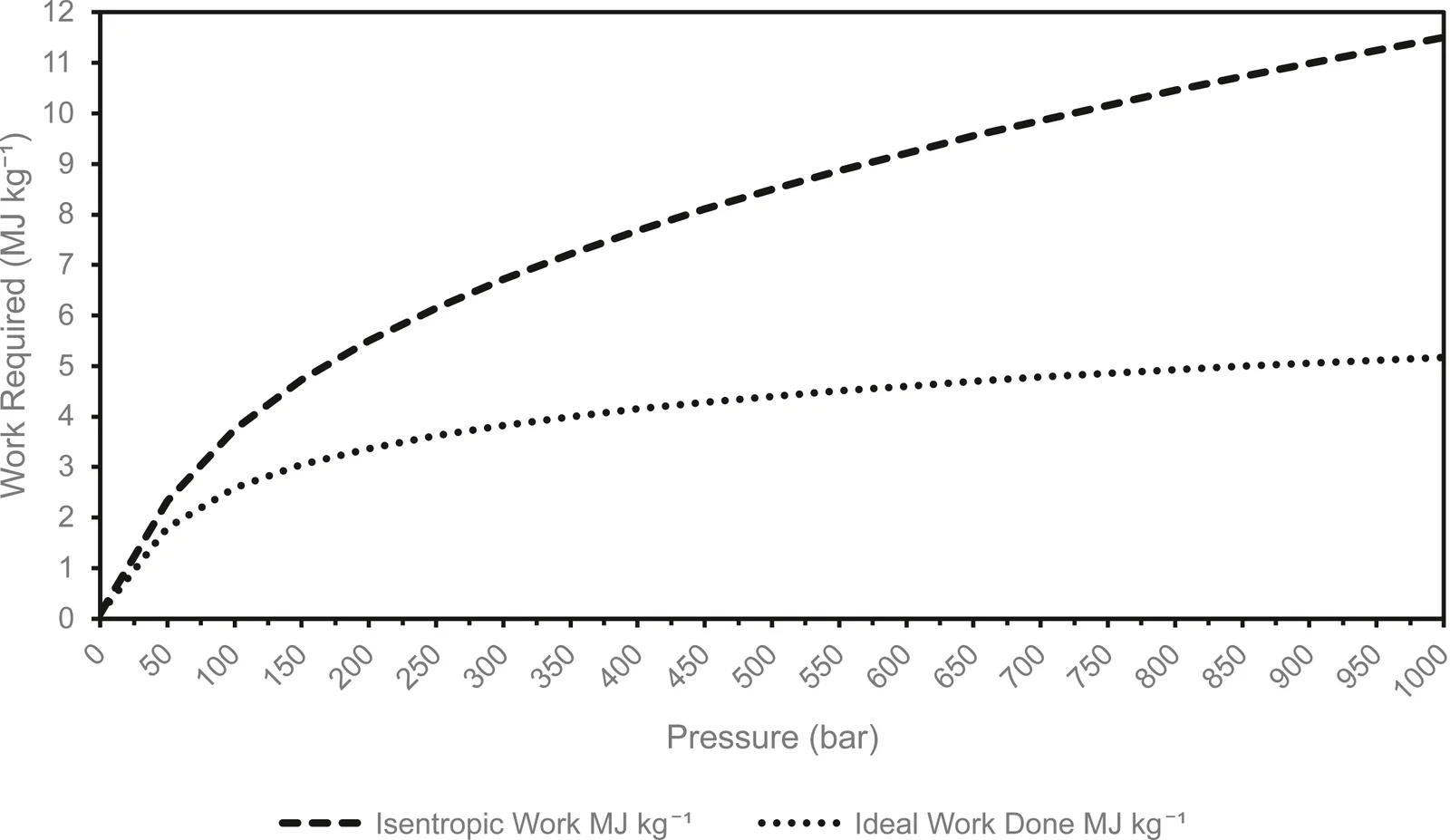
Differences in isentropic compressibility of ideal and real hydrogen at 273.15 K calculated using NIST REFPROP^5^ and the principles of the steady-flow energy equation Of all the gases, hydrogen deviates the most from an ideal gas behavior being heavily influenced by pressure and temperature.

Likewise, thermal management is required both during compression for storage and during refueling. If unabated, temperatures during refueling inside a tank can be upwards of 470 K. This is a result of the compression of gas inside the tank by several hundred bar and the negative Joule-Thompson effect, which dictates that expanding hydrogen at ambient conditions will experience a temperature rise.^6^ Consequently, filling stations often employ a pre-cooling unit to prevent high temperatures from damaging carbon fiber reinforced polymer tanks. Cooling the hydrogen for dispensing down to 233 K requires at least 1.08 MJ kg^−1^ and adds an equipment cost of US$0.50 per kg.^7^ For much slower refueling rates, pre-cooling would not be required^8^ highlighting the trade-off made between vehicle performance, refueling time and station design. Finally, following Charles’s law the final pressure will be lower when the gas cools as it will shrink, the warm gas is less dense leading to fewer moles of hydrogen ultimately being stored inside the vehicle.

These factors are exacerbated by current systems leaving scope for improvement; dispensers typically provide a higher pressure to compensate resulting in delivery pressures in the range of 950 bar.^9^^,^^10^^,^^11^

### Distribution

The method of supplying hydrogen to a refueling station requires careful planning. Unlike natural gas or electricity, there is no national distribution network for hydrogen although gaseous pipelines are being explored as a possibility.^12^^,^^13^ Most stations produce and package hydrogen on-site with an electrolyzer and compressor system^8^; this can simplify logistics greatly by not relying on external deliveries and instead utilize easily accessible grid energy. However, this means that operators must purchase and maintain that equipment for each and every station which is a significant factor to consider. Likewise, benefits from centralized production like waste heat utilization can be underutilized and a reliance on grid electricity exposes the operator to a variable cost of energy that may have been generated using fossil fuels.

Tube trailers delivering to a refueling station via road, while currently uncommon, are envisaged as a popular approach going forward.^8^^,^^11^ Tube trailers contain pressurized hydrogen in one or more vessels affixed to the chassis of the trailer; these must comply with the relevant regulations for road transport limiting their size and weight. Current models made of steel and offer a capacity up to 350 kg at pressures between 200 and 500 bar but offer a low gravimetric density. Composite tanks can allow for much higher delivery pressures and so more efficient distribution by increasing the carried quantity of gas over steel trailers. Currently composite trailers are not commonplace, with high cost being among the reasons they are not widely available. Hydrogen can be liquefied to offer greater distribution efficiency but this requires dedicated plant, specialized trailers and procedures in addition to large quantities of electricity which can render it unfeasible for road use. Compared to a pipeline or on-site electrolyzer, tube trailers can offer a simple and less capital-intensive method of delivering hydrogen if available from a nearby supplier. However, they do not benefit from an economy of scale and some predict that at high utilization it would be unfeasible to supply an HRS via road with current trailer capacities due to the excessive traffic of trailer deliveries required.^14^

### Refueling station design

In order to refill a vehicle today, hydrogen must be rapidly dispensed at rates of several kg a minute, a high-pressure hose is the predominant method of achieving this.^6^ The flexibility afforded by a hose allows for a variety of vehicle types to share a filling station requiring only a compatible connector, in a manner reminiscent of a fossil fuel filling station. The high-pressure hydrogen must be decanted from storage tanks via either a cascade system of increasingly pressurized buffer tanks or directly from a compressor, which must be sized to match the required flow rate for rapid refueling. A buffer tank system can allow multiple vehicles to be supplied at once without needing a larger compressor potentially reducing cost but also adding complexity.^15^

Cost is one of the main issues with current hydrogen refuelling.^16^ In some small scale stations, dispensing equipment adds up to US$6–8 per kg onto the price of fuel.^17^^,^^18^ In addition, evidence gathered from stations across Europe indicates that dispensers, compressors and chiller units were the single largest contributors to station unreliability, responsible for ∼63% of downtime.^19^ This downtime is unacceptable when hydrogen stations are few and far between, highlighting a significant hurdle to hydrogen vehicle adoption. The barriers to hydrogen fuel adoption can be alleviated by increasing energy efficiency and reducing the total cost of ownership (TCoE) of stations, by proxy this then can reduce the cost of fuel to the customer.

Conventional fixed-tank, hose-dispenser refuelling approaches have been in use for decades, being a legacy of fossil-fuels. With gaseous hydrogen refuelling technology only seeing incremental developments in recent years^16^; barriers like cost, scalability and a lack of distribution network remain. In light of this, it is useful to explore an alternative pathway for hydrogen refueling to move past these barriers.

### Swappable refueling

We could apply the learnings from other sustainable fuel transitions as much like with hydrogen, the method of storing energy is the main barrier to battery electric vehicle adoption. To overcome technical limitations, an alternative infrastructure based on battery swapping has emerged as a way to bypass the slow charging times seen in current road vehicles.^20^ This is where packs are replaced and recharged outside a vehicle, Vallera^21^ outlines the key benefits and limitations of battery swapping for road vehicles. Recently deployed systems include the Sun Mobility project in India which reported benefits like a reduction in the time a vehicle was stationary to charge and increased utilization of off-peak electricity.^22^ While hydrogen comes with its own benefits and challenges, if battery packs can be swapped from a vehicle then why not hydrogen tanks?

Therefore, we look to see if swappable hydrogen refueling has been done before in the body of knowledge available to us. In order to provide clarity, we are defining swappable as “standardized interchangeable modular tanks that are designed to be specifically removed and replaced quickly, on a regular basis.” As opposed to “modular” approaches which are not explicitly designed to be removed and replaced or “replaceable” which does not specify how often or easily a storage medium can be replaced. A swappable hydrogen refueling system (SHRS) is defined as refueling infrastructure which prioritizes the exchanging of physical media as a means of refueling over direct gas handling. A literature search was conducted across major repositories including Compendex, Web of Science, Google Scholar, and SAE Mobilus. However, results were scarce when searched using terms including swappable, modular, replaceable, off-board refueling, interchangeable. One notable study, Løvvik^23^ began to describe the outline of a replaceable hydride system with utilization for waste heat but since publication in 2008 there has been no further development. Exploration would still be needed to assess the feasibility of such a system and a method of swapping such proposed storages was not provided, a key challenge would be the short service life seen with current hydrides and developing equipment to suitably recharge in a public setting. Replaceable hydride systems see some minor interest as a way to overcome the long refueling times of metal hydrides, Lieutenant^24^ provides a general review on the state of “off-board” refueling for metal hydrides with a favorable overview for “changeable” solid-state storages citing faster refueling times and system efficiency gains from the use of hydrides.

Meanwhile, in the commercial space a couple of ventures were notable. Toyota recently showcased a portable hydrogen cartridge ecosystem as a “proof of concept” which alluded to being used one day for vehicles.^25^ These are designed to be exchanged by hand but this limits capacity and therefore practicality for vehicles, requiring hundreds to refuel a typical car. In a similar vein, the NAMX hydrogen concept SUV featured removable fuel “capsules”; however, these are advertised as a “secondary” storage.^26^ The vehicle still relies on a primary fixed tank being refueled via pressure hose and is not yet available to the public.

While no truly “swappable” hydrogen refueling system for road vehicles exists publicly, it evident that there is both a commercial and academic appetite for an alternative refueling methods. This article presents a feasibility study into the potential for a swappable refueling system. We have undertaken a technical feasibility study on a conceptual alternative refueling system design. The energy and equipment needs for a swappable station have been analyzed and compared with conventional approaches to assess its viability as a solution to the challenges facing conventional HRS approaches. The swappable system aims to address the issues of efficient distribution and dispensing of hydrogen fuel which are key areas where technological advancement has not kept pace with the rest of the field. While production and end-use of hydrogen are where most energy is utilized, there is already significant investment into these areas and so they are outside the scope of this study.

## Results

### Conceptual design of a swappable system

Given the apparent lack of pre-existing swappable hydrogen systems, a rudimentary concept system is demonstrated for benchmark comparison. The success criteria for a swappable system are as follows.

• The system should not take significantly longer than existing refueling methods.
• It should optimize for cost and energy efficiency to reduce the TCoE.
• The design of swappable vehicles should differ as little as possible from existing automobiles.

The concept is intended to work as illustrated in Figure 2. A vehicle arrives at the station, the used tank is removed and a full tank is inserted into the vehicle completing the refueling process. As the composite tanks are too heavy for a person to lift, a robotic attendant carries the tanks from storage and lifts them into the vehicle. Tanks are stored in movable modular racks, when the rack is empty they can be hauled via trailer to a central facility for refilling. There they can be refilled with hydrogen at the compressor station; this could be situated at the outlet of a hydrogen pipeline or co-located with a hydrogen production facility. Pressure regulation can happen either on the tank or in the vehicle; each offering their own trade-offs.

**Figure 2:**
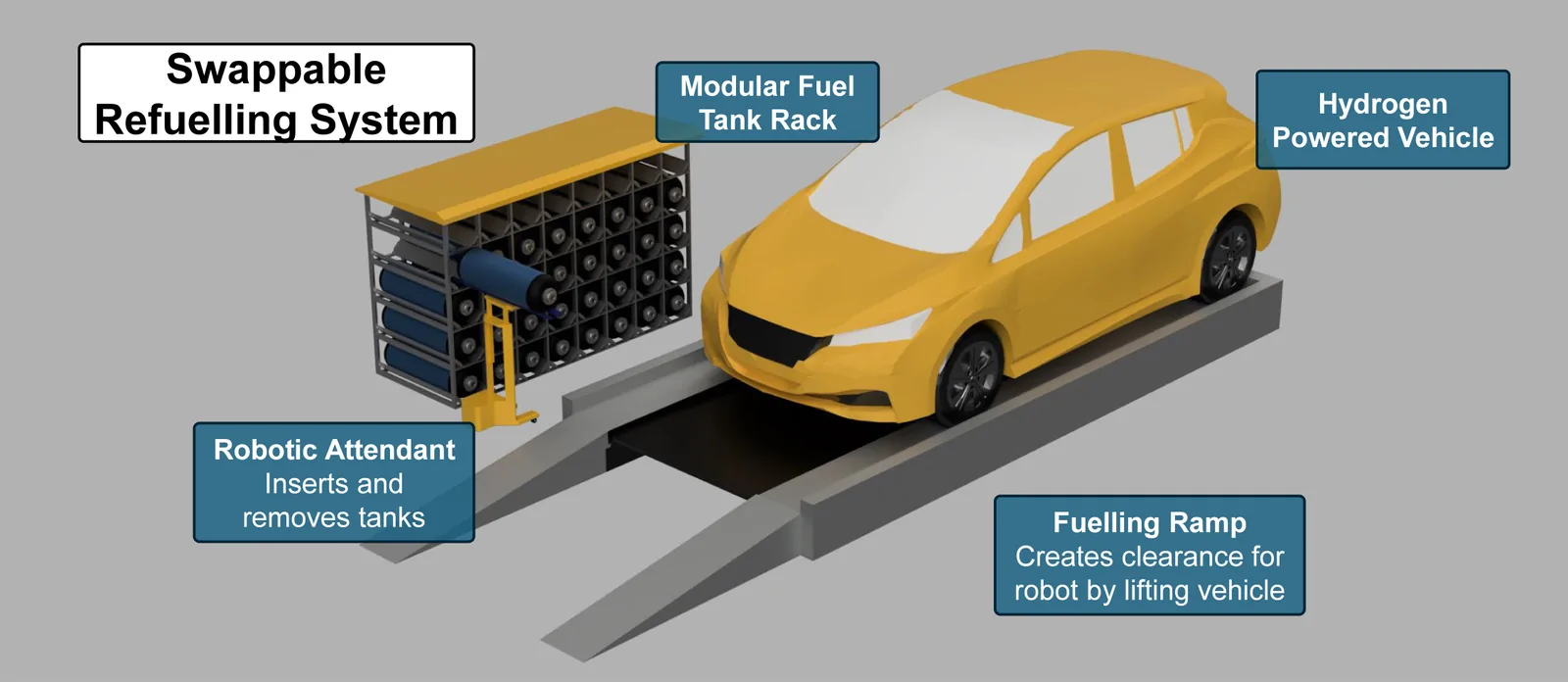
Render of the proposed conceptual swappable hydrogen refuelling station for generic road vehicles highlighting the usage of automated systems to insert and fuel remove tanks from a vehicle

We can outline the assumed performance figures of each distribution mode as done in Tables 1 and 2 to enable comparison between the two systems. It is evident that transporting hydrogen gas at significantly higher pressures can deliver greater efficiency and that composite materials provide superior performance; however, both trailers still suffer from high gross mass which may impede distribution in areas with weight restrictions or poor roads. With this, we can begin to compare the performance of each system in a variety of conditions.

**Table 1:** Hydrogen tank properties from commercial source, with a typical gravimetric efficiency

Swappable tank assumptions:	
System mass:	70 kg
Hydrogen capacity:	4.1 kg
Storage pressure:	700 bar
Outlet pressure:	40 bar
Tank dimensions:	L 1,076 mm Ø 350 mm

The regulator and associated valve system are of insignificant mass and dimensions and so are assumed to be included in this estimation.

**Table 2:** Preliminary performance figures to enable comparison between the proposed road vehicle and current hydrogen transportation methods

SHRS delivery vehicle assumptions:		Conventional tube trailer assumptions:	
Tractor mass:	8,500 kg	Tractor mass:	8,500 kg
Trailer mass:	4,500 kg	Trailer mass:	31,500 kg
Net mass:	29,400 kg	Gross mass:	40,000 kg
Gross mass:	42,400 kg	Storage pressure:	200–500 bar
Storage pressure:	700 bar	Hydrogen capacity:	350 kg
Swap tanks carried:	420	–	–
Hydrogen capacity:	1,722 kg	–	–

As can be seen in Figure 3, the conventional route requires up to three separate compressors for 700 bar cascade refueling and a variety of different buffer vessels. Meanwhile, the swappable route has a central compressor station serving multiple stations and utilizes a single vessel across the entire journey from fuel production to use in the vehicle. This can dramatically simplify the system, cutting down on the number of expensive components across the infrastructure chain and increasing the flexibility of distribution. Both systems have a 4-stage intercooled compressor for efficiency and safety, for the swappable system a compression ratio of 2.5 was used, whereas conventional refueling requires higher pressures and therefore has a ratio of 3 instead.

**Figure 3:**
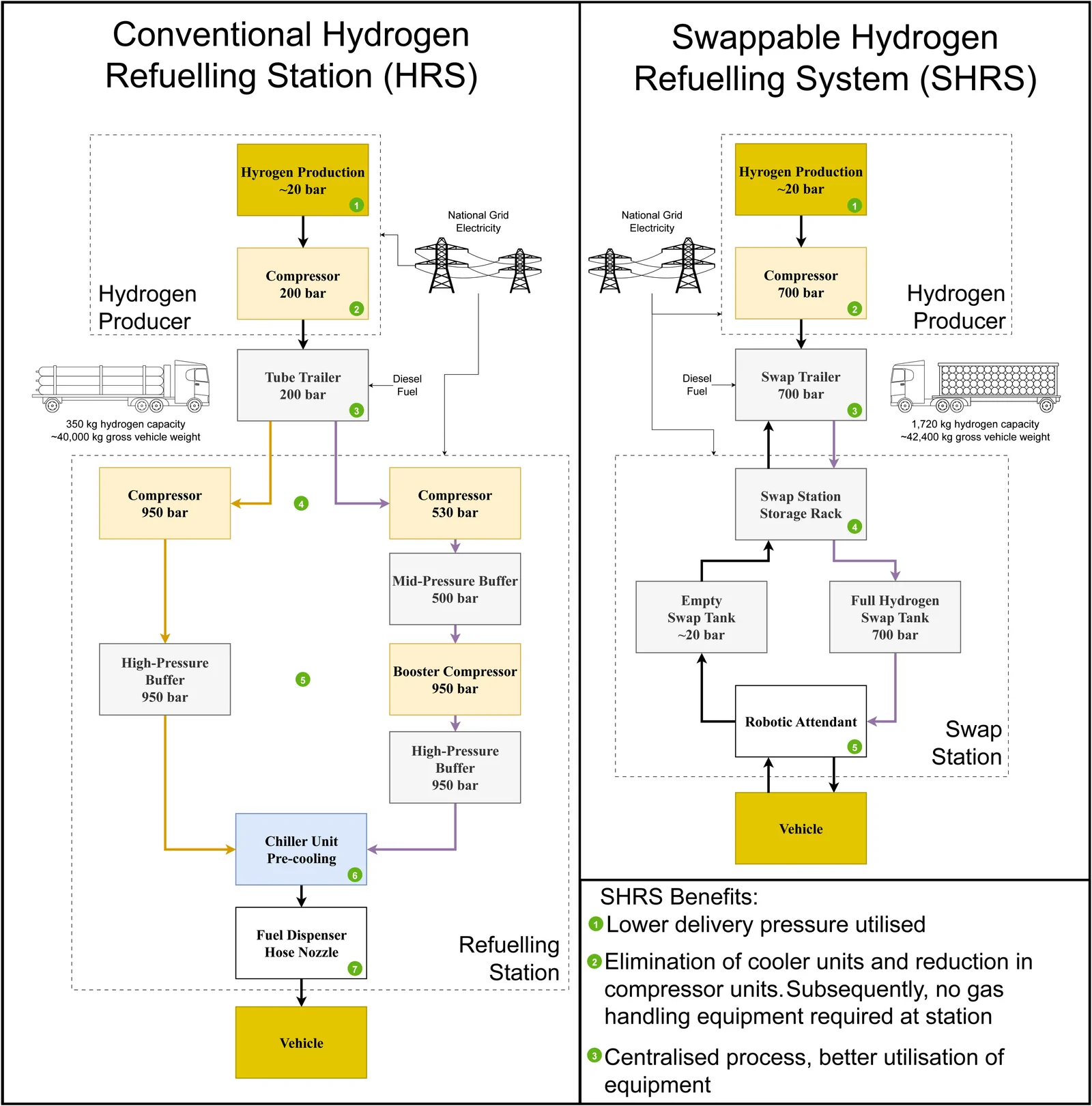
System diagrams for the current and proposed swappable infrastructure system, the key takeaway is a reduction in equipment required to reach the same goal of refuelling the vehicle in an SHRS

### Economic analysis

Estimating the OPEX costs of an infrastructure system can be difficult. However, CAPEX has more information available publicly to enable comparison. Therefore, we have conducted an initial comparison between the systems to understand the cost implications of the different approaches.

When comparing the two refuelling systems in Tables 3 and 4, the SHRS offers a lower CAPEX cost assuming at least five other stations would be constructed. With an SHRS, shared infrastructure would likely provide an economy of scale whereas with a conventional HRS compartmentalization means these benefits would not emerge. The comparison is not ideal as the assumption is made that demand would be at 100% and therefore excess electrolyzer capacity is installed, this leads to an excess of storage tanks in the SHRS as transport was not included. Ideally, the swappable system would rely on more regular refills and more efficient electrolyzer utilization in order to reduce the capital costs on a per station basis leading to lower costs.

**Table 3:** Estimated pricing of a conventional HRS with a medium to large capacity by component, excluding ancillary construction costs such as substructure

Conventional station - 850 kg dispensing capacity			
Item	Units	*Cost per unit*	CAPEX
Compressor	1	*£ 320*,*000*^16^	£ 320,000
Refrigerator	1	*£ 120*,*000*^16^	£ 120,000
Storage	1	*£ 150*,*000*^16^	£ 150,000
Dispenser	2	*£ 67*,*500*^16^	£ 135,000
Electrical	1	*£ 64*,*000*^16^	£ 64,000
Electrolyser	500	*£ 1*,*188*^27^	£ 594,000
–	–	**Total Equipment Cost**	**£ 1,383,000**
–	–	**CAPEX Per kg**	**£ 1,627.06**

**Table 4:** Estimated pricing of an SHRS with a large capacity by component, excluding ancillary construction costs such as storage racking

Swappable station - 853 kg installed capacity			
Item	Units	*Cost per unit*	CAPEX
Robotics	2	*£ 45*,*000*^28^	£ 90,000
Ramp	2	*£ 4*,*000*	£ 8,000
Storage	208	*£ 2*,*050*^29^	£ 426,400
Compressor	0.2	*£ 320*,*000*	£ 64,000
Electrolyser	500	*£ 1*,*188*^27^	£ 594,000
–	–	**Total equipment cost**	**£ 1,182,400**
–	–	**CAPEX per kg**	**£ 1,386.49**

Under this assumption, the cost of the centralized compressor has been spread across five stations owing to more efficient utilization.

### Capacity comparison

Comparing the SHRS with a conventional HRS, evaluation on the possible performance was carried out. Using the typical benchmark capacities for conventional stations, swappable equivalents were created for the purpose of comparison. The conventional HRS characteristics are based on assumptions made from current commercial offerings such as the SANY 580 station,^30^ the calculation for deliveries assumes that 100% capacity is utilized across a week.

For comparative purposes, the number of 4.1 kg hydrogen tanks required for onsite storage at both types of site is given. Each tank would provide a typical passenger vehicle an estimated range of 360 km, based on the fuel consumption of the second-generation Toyota Mirai. Whereas in the SHRS, this also corresponds to the number of tanks stored on site, for the conventional system the hydrogen would typically be stored in a single volume.

The standout figure from Table 5 is the number of deliveries required to provide a weeks’ worth of service with the SHRS requiring far fewer deliveries than the conventional HRS system. The greater quantity of fuel carried per truck trip from the hydrogen production plant in the swappable system results in over five times fewer deliveries per week than its conventional counterpart. This becomes particularly relevant at the largest capacity station, where upwards of 20 tube trailers would arrive in a week equating to roughly three a day, given the complexities of handling hydrogen gas and the resulting noise and traffic disruption this could prove a challenge for a site in an urban center.

**Table 5:** Typical station capacities assuming maximum utilization daily compared across a week

Scenario 1	**Conventional HRS - 150 kg per day**			**Small swap station**	
	Vehicle refuelling points:	2		Vehicle refuelling points:	1
	Daily dispensing Capacity:	150 kg H_2_		Daily dispensing Capacity:	>180 Tanks
	On-site storage Capacity:	∼200 kg H_2_		On-site storage Capacity:	738 kg H_2_
	Hydrogen storage Cylinders on-site:	50 Tanks		Hydrogen storage Cylinders on-site:	180 Tanks
	Tube trailer Deliveries per week:	3 Trailers		Swap trailer Deliveries per week:	0.6 Trailers
Scenario 2	**Conventional HRS - 500 kg per day**			**Medium swap station**	
	Vehicle refuelling points:	4		Vehicle refuelling points:	2
	Daily dispensing Capacity:	<500 kg H_2_		Daily dispensing Capacity:	>360 Tanks
	On-site storage Capacity:	∼500 kg H_2_		On-site storage Capacity:	1,476 kg H_2_
	Hydrogen storage Cylinders on-site:	122 Tanks		Hydrogen storage Cylinders on-site:	360 Tanks
	Tube trailer Deliveries per Wwek:	10 Trailers		Swap trailer Deliveries per week:	2 Trailers
Scenario 3	**Conventional HRS - 1,000 kg per day**			**Large swap station**	
	Vehicle refuelling points:	8		Vehicle refuelling points:	4
	Daily dispensing Capacity:	1,000 kg H_2_		Daily dispensing Capacity:	>420 Tanks
	On-site storage Capacity:	∼1,500 kg H_2_		On-site storage Capacity:	1,050 kg H_2_
	Hydrogen storage Cylinders on-site:	366 Tanks		Hydrogen storage Cylinders on-site:	420
	Tube trailer Deliveries per week:	20 Trailers		Swap trailer Deliveries per week:	4 Trailers
Typical fossil	***Typical fuel station in the UK*, *estimated from DESNZ data***				
	Vehicle refuelling points:		8		
	Typical dispensing amounts (kg fuel day):		12746 (DESNZ)		
	Dispensed amounts converted to H_2_ on LHV basis:		∼4,500 kg H_2_		
	Typical storage capacity (kg):		89,226		
	Cistern trailer (25,000 kg capacity) deliveries per week:		4 Trailers		

The SHRS would only be limited by the time it takes to physically move the tanks and for vehicles to drive on so capacity does not translate well unlike the conventional HRS which is limited by the flow-rates of installed equipment. Fossil fuel data^31^ provided for comparison and shows the difference in capacities typically seen today.

The mass of the swappable trailer was derived by multiplying the mass of the cylinders (70 kg) by the number carried (420), this leads to a payload mass of ∼29,400 kg. This further increases when factoring the mass of tractor unit at 8,000 kg,^32^ with trailer and racking included the gross vehicle weight would be ∼42,400 kg. While specific data for the mass of steel tube trailers is lacking, an estimated gross vehicle weight would be ∼40,000 kg using the same tractor unit. The characteristics of the composite trailer are based on the commercial data listed by Chesterfield special cylinders with a 1,000 kg capacity and mass of 44,000 kg.^33^

### Compression and transportation comparison

To compare the conventional refueling system and the proposed swappable system, the energy required to package and dispense fuel for each pathway is calculated and compared. Greater detail on the calculation method for compression, cooling and transportation energy use along with the assumptions made can be found in the STAR Methods section.

Taking a holistic approach to the delivery and dispensing of hydrogen calculated as a real gas, swappable tanks offer the most energy efficient infrastructure system. Figure 4 shows the different methods of distributing fuel to an HRS, the calculated amount of energy consumed by each is laid out allowing for comparison between each in terms of megajoules used per kilogram of hydrogen dispensed.

**Figure 4:**
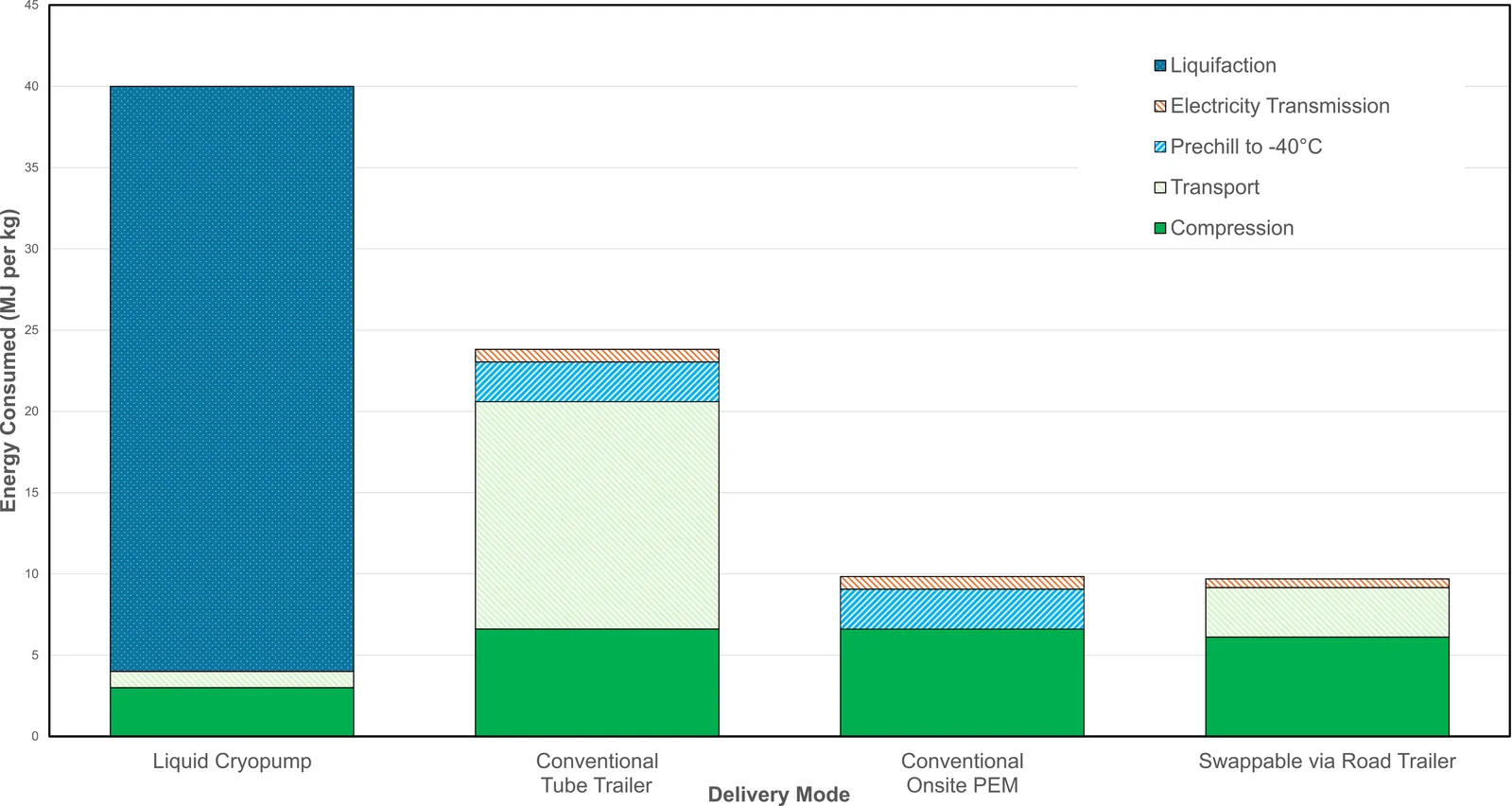
Bar chart showing the calculated energy consumption for delivering 1 kg of hydrogen to a station 100 km away (200 km round trip), for each fuel distribution method Values for liquid hydrogen well-to-tank performance are from the Zemo Report^8^ and are included for comparison.

From Figure 4, we can see that swappable road transport requires around one-fifth of the energy to transport the same quantity of hydrogen as conventional tube trailers. This is due to the fact that both modes have a similar trailer mass thereby requiring the same amount of energy per trip; however, swappable carries far more hydrogen per trailer resulting in a superior overall performance. If hydrogen had to be transported across a significantly greater distance, an on-site electrolyser would likely offer a more energy efficient delivery mode. This is because high voltage distribution offers a more energy efficient method of delivering energy over a road vehicle; across greater distances the network losses from transformation become less significant.

Pre-cooling requirements appear to have a minor impact when compared with the calculated energy required for compression or transportation. The result calculated is closer to a theoretical minimum and will be higher in the real world especially when considering idle energy consumption. Eliminating this step via the swappable method would yield only a minor energy saving although equipment costs are still relevant. Overall, swappable refuelling performed the best under the tested conditions, requiring 9.83 MJ kg^−1^ of energy versus the 23.82 MJ kg^−1^ required for conventional HRS routes representing a saving of 58%.

Our sensitivity analysis in Figures 5, 6, and 7 show that across a range of conditions the swappable system is expected to provide a performance improvement over steel tube trailers. However, at greater distances on-site hydrogen electrolysis may prove more energy efficiency and that at higher capacity levels system performance would be similar. All conditions are baseline except for the variable altered in each calculation.

**Figure 5:**
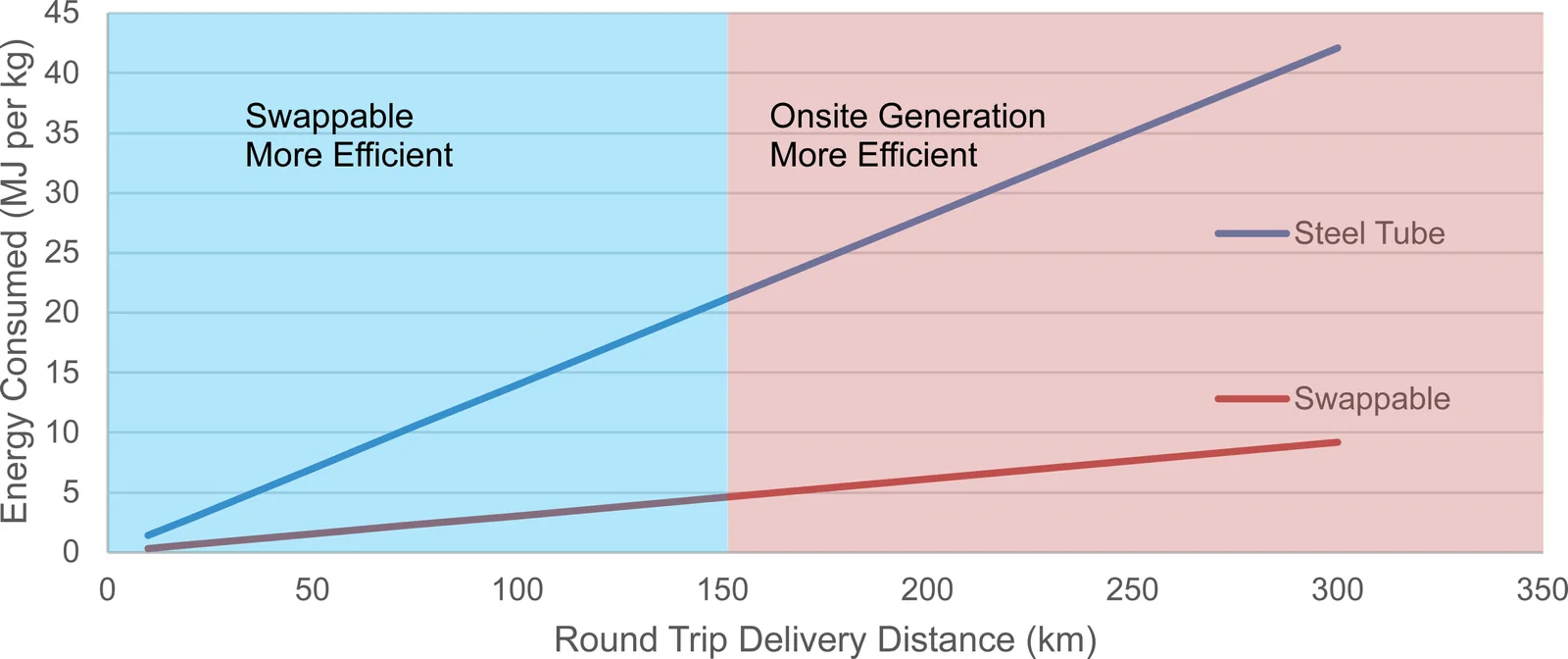
Demonstrates that swappable which becomes significant after at least 50 km of distance from hydrogen supply to the station, past distances of 150 km the transport energy required would make on-site generation more attractive from an energy efficiency standpoint

**Figure 6:**
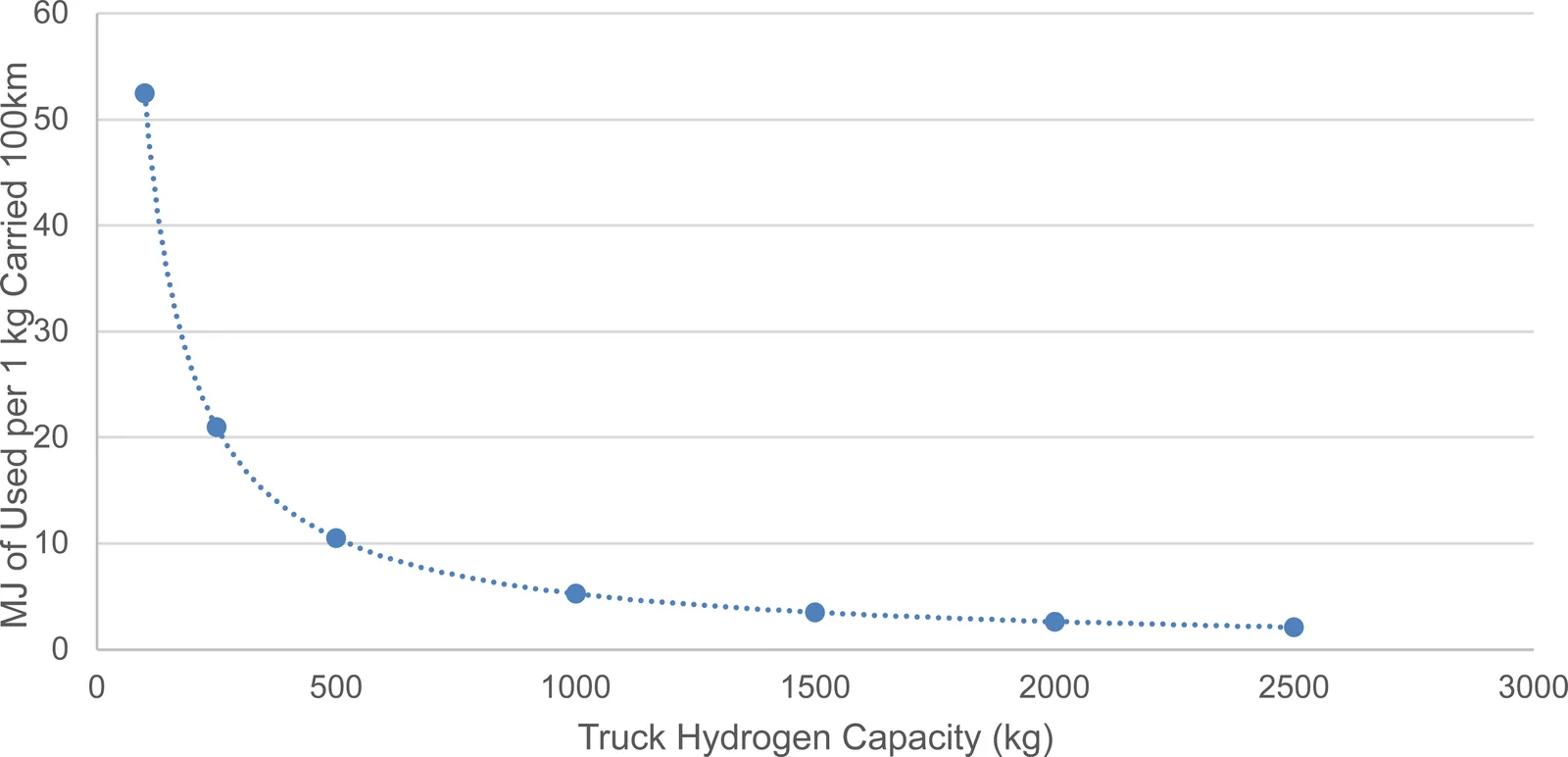
Demonstrates that from an energy efficiency perspective, the fuel capacity of the truck trailer is only significantly relevant at lower capacities

**Figure 7:**
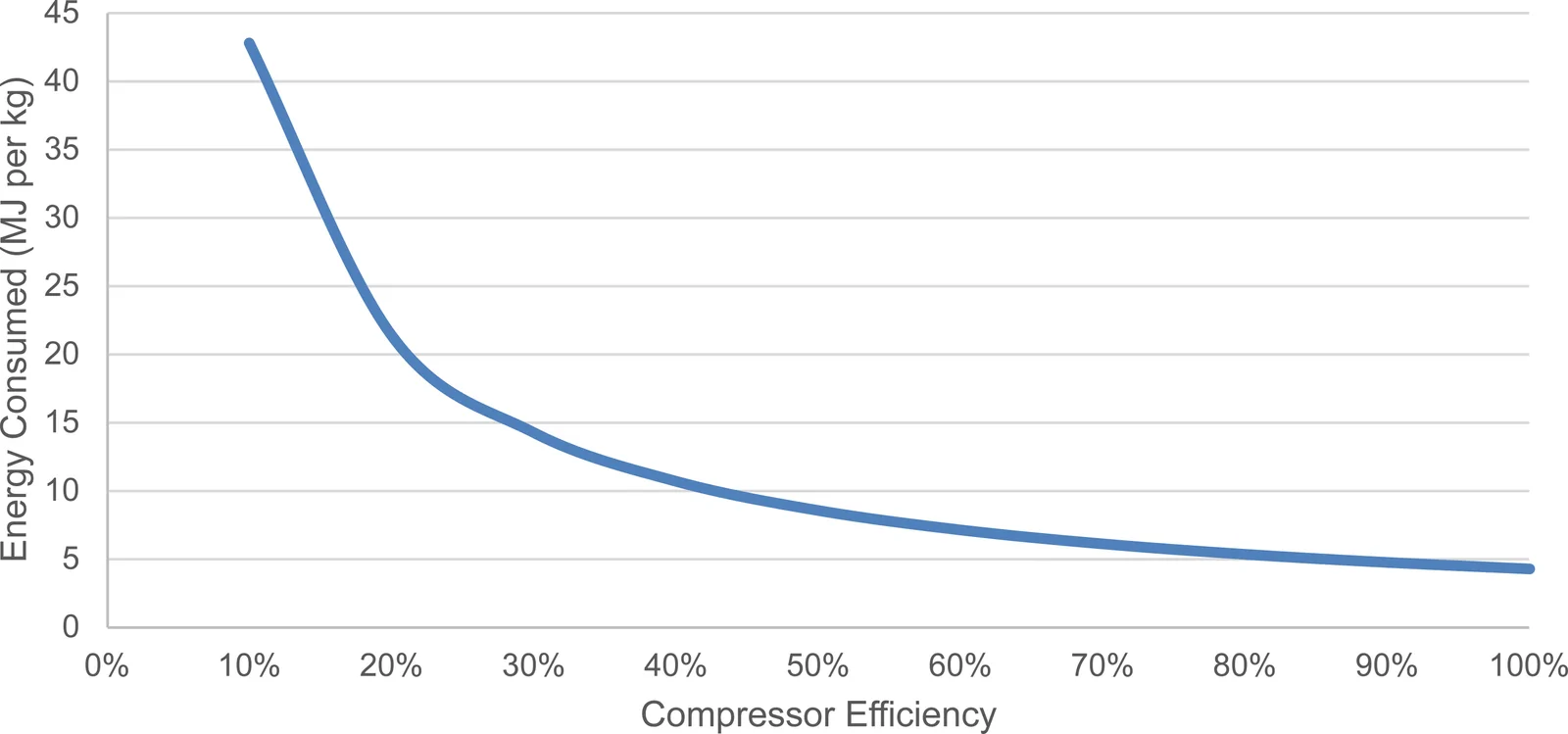
Shows the impact of compression efficiency on distribution energy for the swappable system, across a range of efficiencies this number does not shift drastically under realistic parameters As η represents efficiency as a percentage, when efficiency increases there is an inverse relationship to energy consumed as represented in the chart.

Proposed hydrogen sites in the UK generally sit within 50 km of a major population center; however, this may be relevant for much larger countries like the United States where other distribution methods than swappable may be more competitive.

A more detailed breakdown of the losses in the conventional and swappable systems is presented in the next section.

### Overall well-to-tank energy analysis

The energy used by distribution and dispensing is put into perspective by factoring in a typical upstream energy usage profile. To illustrate the difference between the two systems, Sankey diagrams were created comparing conventional tube trailer and swappable road trailer systems. This additionally includes the energy used to produce the hydrogen to put the distribution and dispensing energy into a wider perspective.

In both Figures 8 and 9, the production of hydrogen is the singular most energy intensive step. With an optimistic proton exchange membrane electrolyzer efficiency of 80%,^34^ 36.34 MJ of energy is unusable for every kilogram of hydrogen produced. Discounting hydrogen’s lower heating value of 120 MJ kg^−1^ which is delivered as useful fuel to the vehicle, the energy “lost” as a result of inefficiency greatly outweighs the cumulative energy needed to deliver and dispense the hydrogen fuel. While a centralized production and refilling system could improve efficiency, this has not been modeled in this study.

**Figure 8:**
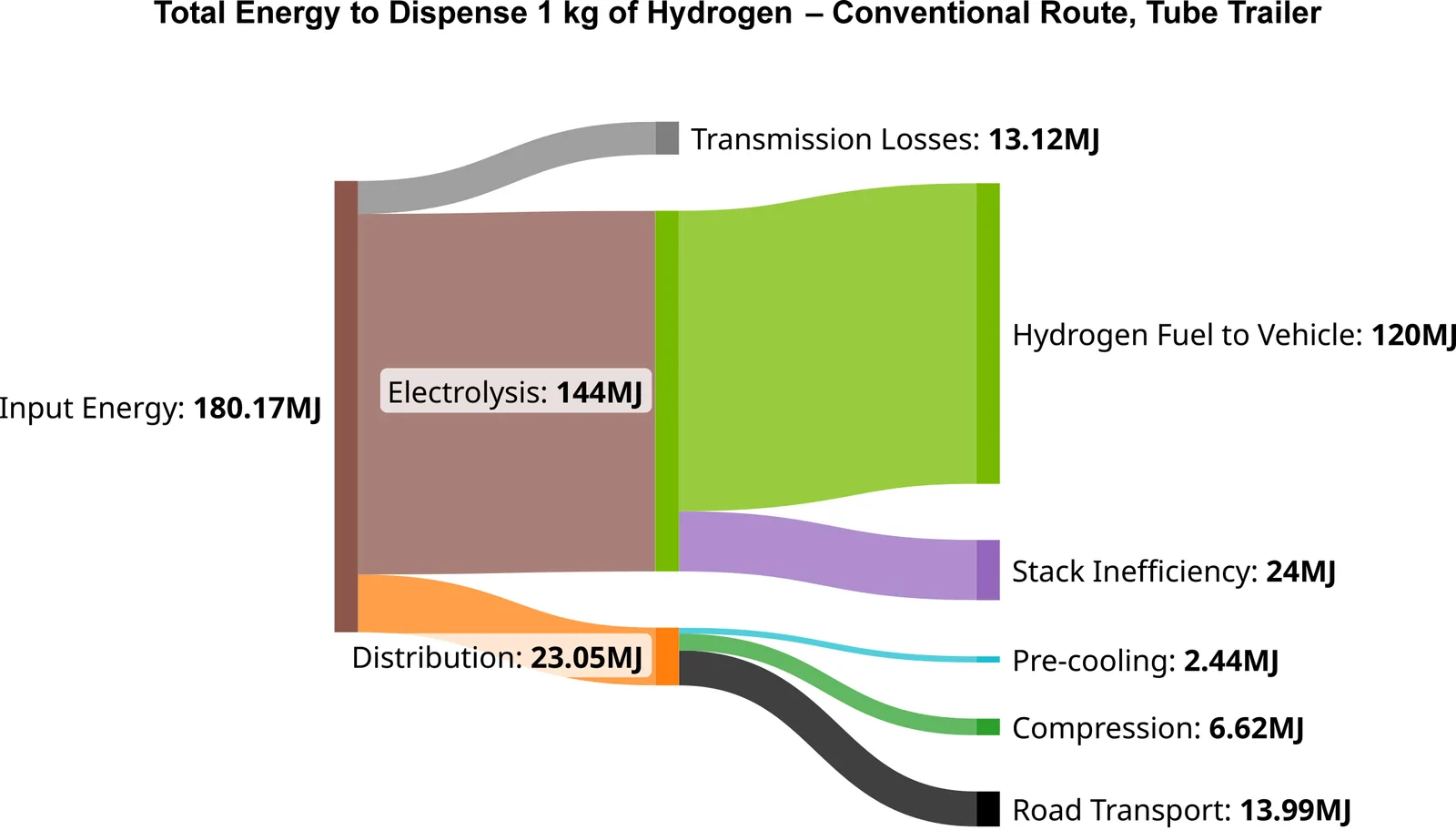
Sankey diagram comparing the energy required to deliver 1 kg of hydrogen across a 200 km round trip, using a hydrogen tube trailer with 350 kg of capacity

**Figure 9:**
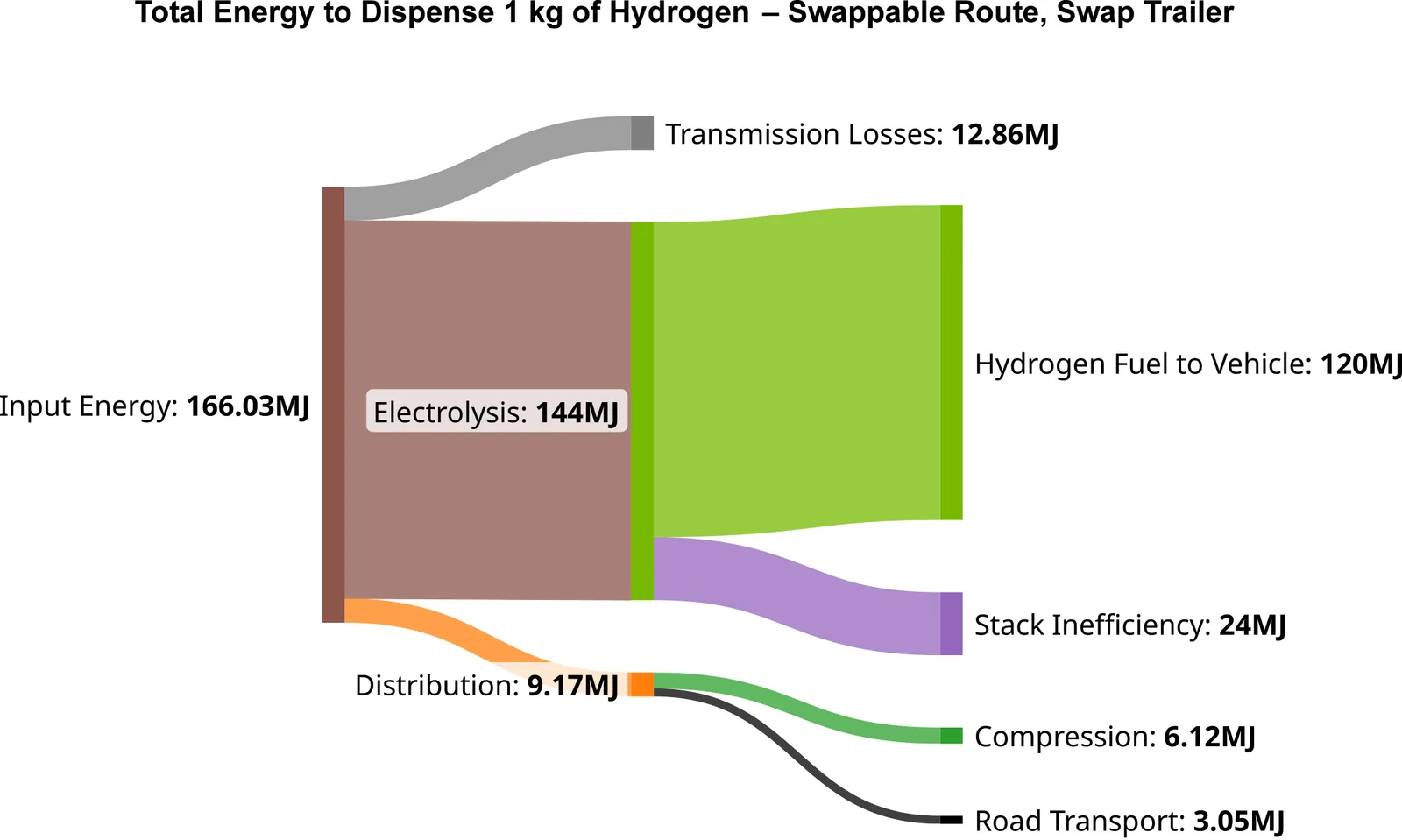
Sankey diagram comparing the energy required to deliver 1 kg of hydrogen across a 200 km round trip, using a swappable hydrogen modular trailer with 1,722 kg capacity

It should be noted that using less fuel for road transport has a greater impact beyond energy use, most trucks on the road today use fossil fuels so reducing fuel usage directly reduces CO_2_ emissions unlike hydrogen production and compression which can more readily use renewable energy sources. This is represented in Figure 10A where using current energy systems there are still emissions from hydrogen fuel, in the swappable system the vast majority of emissions come from diesel combustion for road transport as shown in Figure 10B.

**Figure 10:**
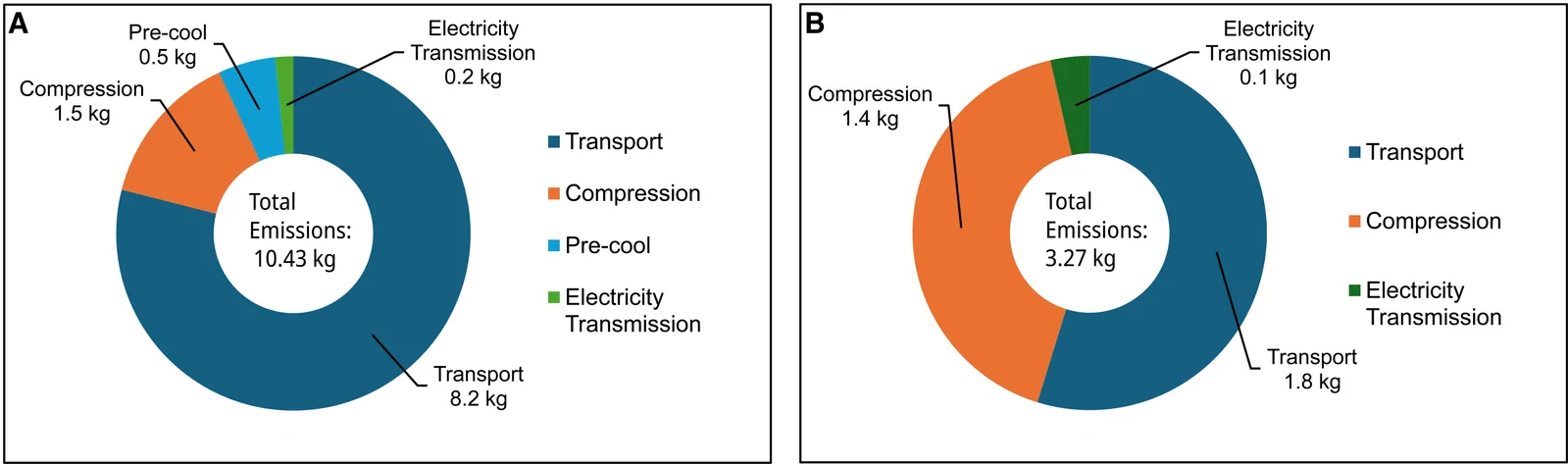
CO_2_ equivalent emissions from energy to dispense a 4.1 kg refuel, chart 1a shows the conventional approach whereas chart 1b shows the swappable route Both were calculated across a 200 km round trip using road diesel blend^35^ for transport and grid electricity^36^ for equipment, multiplied against the calculated energy consumption for each route. In the future, sustainably fueled vehicles are expected to become the norm and so the gap between the two routes will diminish.

Ultimately, the swappable approach brings notable benefits, but production energy is still the dominating factor highlighting the importance of producing hydrogen in a sustainable manner. Although, infrastructure performance does still have a notable impact especially when multiplied across the lifetime of the distribution network.

## Discussion

### Distribution conventional HRS versus swappable

The findings demonstrate that it is evident that swappable hydrogen could bring many benefits to a future hydrogen economy over conventional hose refueling. Conventional hydrogen refueling via pressure hose faces many challenges that have stifled its adoption into the mainstream market. It is imperative to provide a reduction to the TCoE in order to make fuel more affordable for road users and investment more attractive for infrastructure operators. Cutting down on energy usage and slashing the capital investment required to build hydrogen infrastructure provides an avenue for this to happen.

Safe, reliable and cost-effective distribution of fuel is critical to ensuring the development of a hydrogen economy. Given that refueling stations are anticipated to eventually dispense hundreds of kg of hydrogen a week, even small efficiency gains are significant as they are multiplied throughout the supply chain.

The implementation of a fully swappable system has the potential to directly reduce the energy required to deliver hydrogen to an SHRS by up to 58% under the assumed conditions. This potential saving is enormous when multiplied across the lifetime of a nationwide fleet with multiple refueling stations which can directly translate to a lower cost of fuel to road users and a more attractive business proposition to infrastructure operators. It is expected that the real-world performance figures will likely have an even greater gap between the two systems. Factors like electolyzer duty-cycles, heating during compression and chiller unit overhead cooling will likely increase the energy requirements of a conventional HRS network.^7^ Swappable systems are less likely to suffer from such inefficiencies, having both an economy of scale advantage and a greater flexibility in how to operate their equipment by not being required to chill or compress on demand.

By far, the biggest impact was due to higher energy density transporting hydrogen gas from the production facility to the refueling station. As seen in Table 2, a greater payload of hydrogen is delivered per truck trip. This indirectly reduces the CO_2_ emissions of the hydrogen fuel through the reduction in diesel fuel for truck transport as seen in Chart 1 and would likely reduce the cost of fuel by lowering the number of drivers and trucks required. It should be noted that a swappable system is not required for this, and it highlights the benefits of high-pressure storage for transportation for tube trailers. However, the manufacture of large bespoke Type-IV composite hydrogen tanks specifically for transport is prohibitively expensive. Therefore, a swappable approach acts as a key enabler for this technology by transporting the hydrogen in multiple smaller tanks which can be manufactured at scale. Our results demonstrate that this approach still produces significant benefit over conventional tube trailers.

Finally, the results show that the direct losses as a result of lower peak pressure requirement (0.54 MJ kg^−1^) and no need for pre-chilling (2.44 MJ kg^−1^) represent a saving of approximately 1.7% of the overall energy required to create and dispense hydrogen fuel.

### Station design

Optimizing station performance is critical when looking to scale up infrastructure, reducing the total cost of ownership is vital to enabling a widespread distribution network to exist.

The compressor system is a prime target for improvement, being by far the most expensive component of an HRS and easily exceeding US$1M for a 500 kg per day station.^10^ Meanwhile, swappable refueling eliminates the need for compression at refueling stations. Having a shared central compression facility allows for greater utilization rates for a given compressor capacity, presenting the opportunity for significant cost savings. Likewise, removing the need for a chiller or on-site gas production system eliminates most of the cost associated with an HRS. This is assuming that the adaptation of storage and transport equipment for an SHRS does not incur significant cost. Likewise, the SHRS benefits from an economy of scale and therefore the conventional approach may be preferable for limited deployments. As no gas handling is occurring on-site, swappable should be comparatively simpler to operate requiring less frequent maintenance and fewer downtimes. With hydrogen production and compression occurring at scale in close proximity, recovery of waste heat would be practical which may improve the overall energy efficiency of the system.

On-site electrolyzers risk underutilization and eliminating them can bring other benefits. A centralized hydrogen packaging facility can make better use of renewable electricity and even operate “behind the meter” private connections bypassing grid constraints. Intermittent renewables could provide low-cost and low-carbon energy during hours of high-curtailment in areas where energy is plentiful but capacity to transport is limited, this is further discussed by studied around urban microgrids^37^ and co-located renewables^38^ highlighting the benefits of such an approach.

Swappable hydrogen is not without its downsides, notably requiring the standardization of future vehicles and refuelling systems across automakers which may prove too ambitious. To facilitate refueling there must be *“n+1”* fuel tanks in circulation, with tanks sat in the station in addition to inside a vehicle. This may pose a major barrier with the current high unit cost and the fact that carbon-fibre composite tanks have a limited service-life with minimum recyclability. Similarly, methods of handling tanks must be robust in order to avoid costly damage to safety critical components like tanks and fittings with equipment to do this not being commercially available at the time of writing. While handling methods need to be developed, automotive fuel tanks in circulation already exceed requirements for safety standards and existing hardware may only require minor modification. Quick connect pressure fittings designed for repeat usage and user safety are already in use at refueling stations and could be readily adapted onto swappable tanks. In a swappable system, non-destructive testing could be done on tanks out of the vehicle at regular intervals compared to relying on periodic vehicle inspections where specialist skills and equipment may be limited. The hydrogen vehicle space is rapidly developing with regulation evolving in response, the aim of this study is to assess the feasibility of a swappable system but a real-world implementation would need to work alongside regulators to ensure safety and compliance.

In this study it is assumed that only full tanks are supplied and empty tanks are returned. In reality, a fuel tank is unlikely to be fully emptied by the user. While poor tank utilization would reduce system efficiency, the gap in efficiency between tube trailers and swappable is significant enough that this is unlikely to undermine the overall improvement. With this in mind, it is important for large-scale applications to have methods of maximizing tank utilization to increase efficiency and reduce cost especially with regards to the number of tanks in circulation. Such methods could include smart algorithms matching partially filled tanks to driver journeys or incentivizing drivers to only swap tanks when needed. In future work, it would be interesting to explore the break-even point alongside driver behavior and how the number of stations available to a user influences swapping behavior as a future *trans*-disciplinary project. A high cost of tanks could prove a major drawback to the system due to the greater number required but on the other hand, combining a greater storage density and a smaller number of refueling points required means a swap station may be more compact than an existing refueling station. In a “tank as a service system,” minimizing the number in circulation and maximizing their effective lifespan would be paramount to keep TCoE manageable.

Overall, swappable hydrogen represents the opportunity to have a marked improvement in performance over current approaches with further development. We will carry out future work to understand the life cycle and cost implications of these issues to provide a complete technoeconomic assessment. Validation from a prototype system would be required to demonstrate these findings with more certainty and allow for greater insights into how an SHRS could operate.

### Limitations of the study

The findings from this study must be considered in the context of the application and environment as it is based on a conceptual system without real-world prototype validation. While the proposed swappable method offers an efficiency improvement over current standards, this advantage is most pronounced within a specific range of operating scenarios. At shorter travel distances or if more advanced cooling technologies emerge, the relative benefit of the swappable system may diminish. Additionally, the efficiency estimates presented here rely on simplifying assumptions that do not fully capture the diversity of real-world operating conditions or the broad range of system components used in practice, whose performance can vary considerably such as under low-duty cycle operation. To account for this uncertainty, the efficiency estimates adopted throughout this study were deliberately conservative.

## Resource availability

### Lead contact

Further information and requests for resources should be directed to and will be fulfilled by the lead contact, William Forshaw (wf374@bath.ac.uk).

### Materials availability

This study analyses existing, publicly available data.

### Data and code availability

• Data reported in this study will be shared by the lead contact upon reasonable request.
• This study does not report original code.
• Any additional information required to reanalyze the data reported in this study is available from the lead contact upon request.

## Acknowledgments

This project was supported by a scholarship from the 10.13039/501100000266EPSRC Center for Doctoral Training in Advanced Automotive Propulsion Systems (AAPS), under the project WP/S023364/1. With thanks to the support of staff at the University of Bath for facilitating the publication of this study.

## Author contributions

Conceptualization, investigation, methodology, and writing – original draft, W.F.; supervision, validation, and editing, T.F., A.H., and T.M.

## Declaration of interests

The authors declare no competing interests.

## STAR★Methods

### Key resources table

**Table:** 

REAGENT or RESOURCE	SOURCE	IDENTIFIER
**Software and algorithms**		

REFPROP - Thermophysical Database	NIST^5^	https://www.nist.gov/srd/refprop

### Method details

In order to determine the characteristics and performance of both conventional and swappable systems a quantitative approach has been taken to understand energy efficiency. Component performance characteristics use reported operational data where possible, where unavailable theoretical calculations are used to estimate these.

#### Thermo-physical properties

Calculations on the thermo-physical properties of hydrogen as a real-gas were carried out. Adiabatic compression was chosen as it provides an appropriate level of accuracy even if overestimation could be possible compared to a polytropic approach. The derived formula for adiabatic compression is explained below:

$$W=\left(h_{2}-h_{1\;}\right)/\eta$$

Where:

$$h_{1}=h\left(T_{1},P_{1}\right)$$

$$s_{1}=s\left(T_{1},P_{1}\right)h_{2}s=h\left(P_{2},s_{1}\right)$$

Where Work *W* represents the energy required for compression in Joules. The efficiency of the compressor system, *η* adjusts for real-world performance and has been set at 70 %^39^ for both systems. The enthalpy of hydrogen in Joules is represented by *h*_*1*_ and *h*_*2*_ are the initial and final respectively. Entropy in J K^-1^ is represented by *s*, while *T* is temperature in Kelvin and *P* is pressure in Pascal. The NIST REFPROP V10^5^ software evaluates thermodynamic properties using Helmholtz-energy–form equations of state and provides outputs that are validated against experimental data. Using the equation of state model from this package we can calculate the specific enthalpy and entropy of pure hydrogen as a real gas under the specified conditions. From the starting pressure of 20 bar (electolyser outlet pressures are commonly 20-40 bar^11^^,^^16^) we can use a typical compression ratio of 2x to 4x that will give us the target pressure at the next compression step. This can be used then to evaluate the enthalpy and entropy at the inlet and outlet as well as the temperature at the target pressure, comparing the enthalpy change calculates the compressor work carried out as derived from the first and second thermodynamic laws. An initial standard temperature of 298.15 K was used, as it is typical for the gas to be above ambient temperature inside gas handling equipment. It is assumed that an intercooler is used between stages to return the gas to 320 K in order to keep outlet temperatures below 475 K which is a typical temperature limit for hydrogen compressor seals, this also will make the compression approximately twice as efficient as without intercooling. The compressibility factor *Z* is included in the Helmholtz equation of state, *Z* grows at an accelerating rate as pressure increases which at high pressures can significantly alter the compression characteristics. In many instances *Z* is excluded for simplicity and some sources recognise that real world figures would be much higher.^9^

The heat to be removed during for pre-cooling was calculated using the sensible heat equation:

Q=m∗cp∗ΔT

Where *Q* is the heat removed in kilojoules. *M* is the mass of substance, 1 kg for all calculations. Δ*T* is the change in temperature in Kelvin, it was assumed the outlet temperature from the final stage of the compressor was 468.61 K and the target temperature to cool to was 233 K following current standards for HRS pre-cooling. While specific heat capacity, *cp* is based on state; a constant of 14.5 kJ kg^-1^ K^-1^ was calculated using REFPROP^5^ for the on-site compressor outlet temperature and pressure. A constant was chosen for simplicity as the decreased accuracy over a differential equation does not significantly alter the final results.

This can be used to calculate the electrical energy consumed by the cooling load:

$$E=\frac{Q}{CoP}$$

Where *E* is the electrical energy required in kilojoules. The coefficient of performance (*CoP*) of the chiller unit was 1.42,^40^ this is unitless and represents the ratio between energy input and useful energy output in a system.

The calculated figure of *E* of 2.44 MJ kg^-1^ is larger due to a smaller coefficient of performance but remains in a sensible range of the 1.08 MJ kg^-1^ quoted by some sources,^7^ both figures are expected to be much higher in the real world due to poor equipment utilisation.^8^ The overall energy to dispense hydrogen conventionally was calculated in this paper to be 9.83 MJ kg^-1^ and is comparable to the 9.85 MJ kg^-1^ estimate reported in the JEC Well-to-Tank report v5^11^, the discrepancy is primarily due to the report not accounting for high voltage distribution and a different final working pressure though both deliver the same 700 bar hydrogen fuel output.

#### Hydrogen storage

The properties of the tube trailer are based on a generalised scenario, reliable reference data on performance is not widely available so an estimation has been made based on given capacity from a variety of sources^41^^,^^42^ in relation to mass and size of trailer.

The Type-IV hydrogen tank chosen as a reference unit is available of the shelf today and is currently in use within the Hyundai Nexo FCEV. It is produced by Iljin HySolus and has partial reference data available from the manufacturer including dimensions and capacity.^43^ However, the mass of the unit was not provided therefore it has been estimated using the provided dimensions and an estimated density of carbon fibre composite. The estimated mass of 70 kg is well within the expected range for an automotive cylinder with a calculated gravimetric storage density of 5.86 %.

#### Fuel distribution

For distribution, a 200 km round trip was chosen as the typical distance many major cities in the UK would be from proposed hydrogen clusters via road. Diesel tractor units are assumed to be used on typical UK roads, the calculated fuel consumption figure of 13.99 MJ kg^-1^ for the tube trailer fall in line with that of other sources such as the Zemo Report at 11 MJ kg^-1^ of hydrogen transported.^8^

The formula for estimating fuel consumption of truck and trailer and may be written as:

Fuelperdistance=Basefuelconsumption−Permassadditionalconsumption∗Mass

The source provides the base fuel consumption at 8.5 mpg for the tractor unit with a reduction of 0.112 mpg per tonne of mass carried.^32^ This source has been chosen as it provides payload-specific fuel consumption figures that take into account typical driving conditions of freight vehicles common in the UK. These can be converted into SI units estimating 3.78 kg of diesel per imperial gallon and a lower heating value for diesel of 44 MJ kg^-1^. This can be fed into the following formula:

Megajoulesoffuelconsumedperunitdelivered=Hydrogencapacity/(Dieseldensity∗LHV∗(Distance/Fuelperdistance))

For the simplicity of calculation, conventional stations are assumed to utilise this same tank as stationary storage. In practice this would not be the case with a HRS cascade system featuring a variety of low, medium and high-pressure buffer storage and possibly a bulk storage tank or stationary tube trailer for further capacity. This approach can be justified in this comparison however, as the cost and materials of the fixed storage likely equate to a similar amount to the equivalent quantity of swappable tanks which store a greater quantity of fuel than the lower pressure fixed tanks in a conventional HRS for a given size.

Losses resulting from grid transmission are treated as an additional energy input and are calculated by taking the electrical energy required to deliver hydrogen and multiplying it by the transmission loss percentage. Losses are estimated at 8.57 %^44^ and for simplicity includes both average network transformation and distribution losses irrespective of distance.

### Quantification and statistical analysis

Parameters are reported as they appear in the paper. The data yielded by the methods utilised met the assumptions of the statistical approach.

For the sensitivity analysis shown in Figure 5, delivery distances showed a linear trend as fuel efficiency factors like engine efficiency and aerodynamic performance are not dependent upon travel distance. A distance of up to 350 km was analysed as it is unlikely for delivery routes to carry gaseous fuel across any greater distance on a regular basis.

For the analysis in Figure 6, the deterministic model shows the expected inverse relationship where energy usage decreases as fuel capacity increases. This does assume all other variables remain constant including a consistent mass of vehicle however, in the real world as current regulations mean that it is however likely for vehicle mass to remain constant. This is likewise with Figure 7, other variables were kept consistent and only efficiency was altered.
